# Detection of hydroacoustic signals on a fiber-optic submarine cable

**DOI:** 10.1038/s41598-021-82093-8

**Published:** 2021-02-02

**Authors:** Hiroyuki Matsumoto, Eiichiro Araki, Toshinori Kimura, Gou Fujie, Kazuya Shiraishi, Takashi Tonegawa, Koichiro Obana, Ryuta Arai, Yuka Kaiho, Yasuyuki Nakamura, Takashi Yokobiki, Shuichi Kodaira, Narumi Takahashi, Robert Ellwood, Victor Yartsev, Martin Karrenbach

**Affiliations:** 1 Japan Agency for Marine-Earth Science and Technology (JAMSTEC), Yokosuka, Japan; 2 National Research Institute for Earth Science and Disaster Resilience (NIED), Tsukuba, Japan; 3 OptaSense Ltd, Farnborough, UK

**Keywords:** Ocean sciences, Solid Earth sciences

## Abstract

A ship-based seismic survey was conducted close to a fiber-optic submarine cable, and 50 km-long distributed acoustic sensing (DAS) recordings with air-gun shots were obtained for the first time. We examine the acquired DAS dataset together with the co-located hydrophones to investigate the detection capability of underwater acoustic (hydroacoustic) signals. Here, we show the hydroacoustic signals identified by the DAS measurement characterizing in frequency-time space. The DAS measurement can be sensitive for hydroacoustic signals in a frequency range from $10^{-1}\,\text {Hz}$ to a few tens of Hz which is similar to the hydrophones. The observed phases of hydroacoustic signals are coherent within a few kilometers along the submarine cable, suggesting the DAS is suitable for applying correlation analysis using hydroacoustic signals. Although our study suggests that virtual sensor’s self-noise of the present DAS measurement is relatively high compared to the conventional in-situ hydroacoustic sensors above a few Hz, the DAS identifies the ocean microseismic background noise along the entire submarine cable except for some cable sections de-coupled from the seafloor.

## Introduction

Various previous studies have shown that a fiber-optic cable itself can be a sensor because optical light scattering effects are relatively sensitive to external influences such as strain, temperature or magnetic field^1–5^. Distributed acoustic sensing (DAS) based on Rayleigh back-scattering of light is one of the fiber-optic sensing technologies enabling us to monitor vibration of the ground by measuring phase changes within a fiber-optic cable due to its strain changes caused by elongation and compression of the fiber^6^. Therefore, DAS functions as spatio-temporally continuous strain sensors deployed from the optical source over a distance of few tens of kilometers. It has been studied that DAS based on Rayleigh back-scattering of light is capable of long-range sensing within a wide frequency range, but with less spatial resolution, as compared to Brillouin based sensing^7^. On the other hand, Brillouin based sensing is commonly employed in distributed strain sensing (DSS) or distributed temperature sensing (DTS), because even ambient condition directly affects the back-scattering of light. However, Brillouin based sensing can suffer from issues of narrow frequency bands, small frequency shift, and reduced sensitivity to temperature. Detailed discussions on the difference in the scattering processes between Rayleigh and Brillouin can be found in an article^8^. The sensor channel spacing along a fiber-optic cable can be decreased down to a few centimeters and the gauge length for each sensor can be controlled independently. The ultimate resolution is controlled by a combination of channel spacing and gauge length. DAS can be regarded as a continuous strain sensor array deployed along a fiber-optic cable. DAS technology, in the early period of its history, has been developed primarily by industry for surveillance of underground reservoirs of oil or gas^9,10^, as well as condition monitoring of pipeline distribution systems^11^. To a lesser degree, DAS technology has been employed in civil engineering for the purpose of monitoring the maintenance requirements and degradation of linear structures such as bridges, railways, embankments or buildings in which material properties are changing over time^12–15^.

Recently DAS technology has been applied to studies of earth science in both on-land and under-sea regions and successfully detected seismic signals. For example, the DAS technique was applied to the on-land fiber-optic cable, and it detected surface waves that originated from regional to global teleseismic events^16–19^, an ice-quake event inside the glacier terrain^20^, and ice sheet displacement^21^. DAS can estimate subsurface structures using artificially controlled sources or ambient noise^22,23^. Previous field studies for earthquake observations and laboratory experiments suggest that DAS data shows the same sensitivity as a seismometer^24^. Some successful applications of DAS technique using a submarine fiber-optic cable have been conducted^25–29^. For example, DAS identified an unknown fault system in Monterey Bay, California U.S.A., as well as a signal originated by a local earthquake nearby^26^. Another seafloor experiment demonstrated that DAS is capable of detecting surface gravity wave with same frequency range as a tsunami in the ocean^28^. These applications of in-situ DAS geophysical measurement were primarily based on passive sources^30^. Additionally, hydro-pressure sensing by DAS has not been common, so only a few literatures can be found^31,32^.

**Figure 1:**
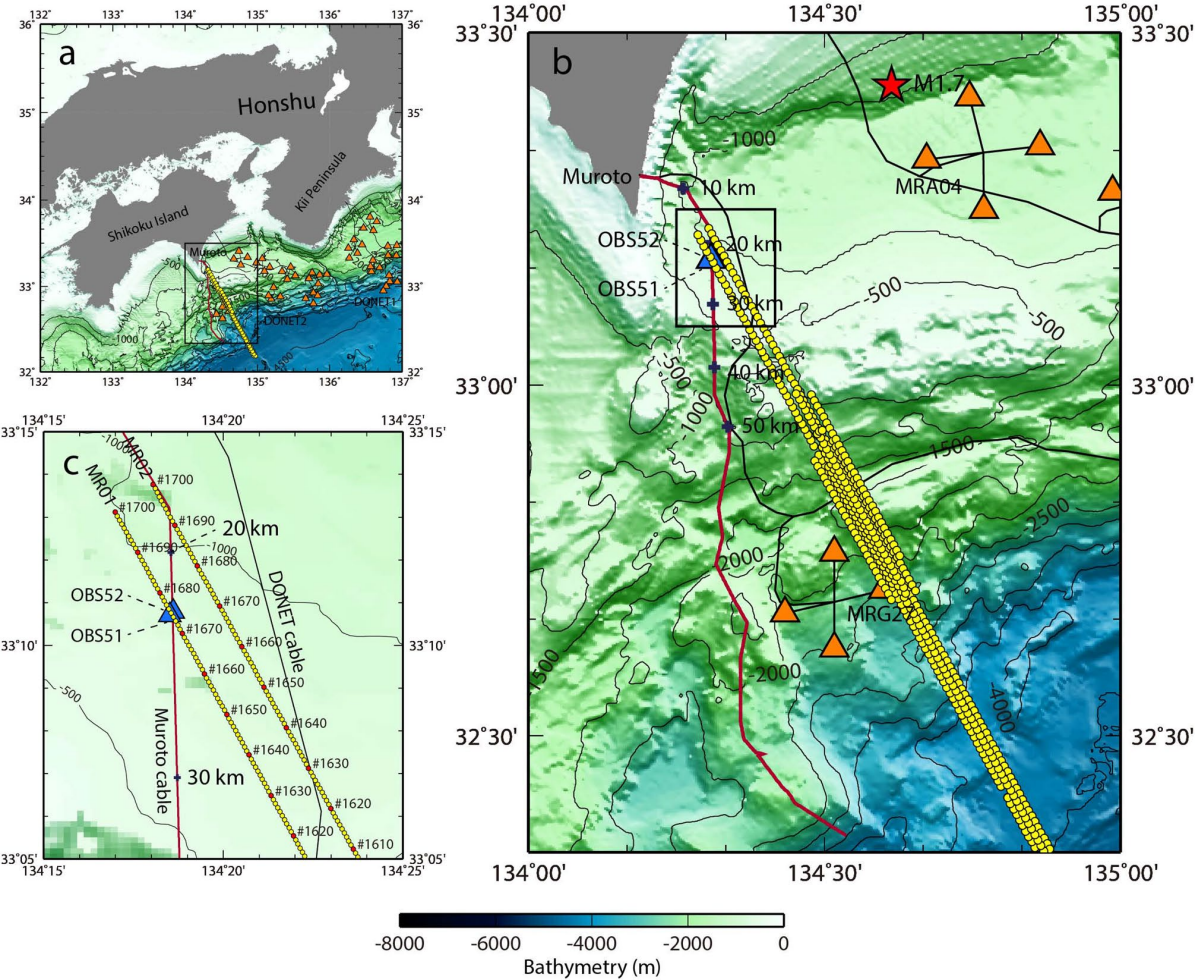
The DAS experimental areas covered by the study. The locations of the Muroto cable (red line), the air-gun shot sites (yellow circles), two OBSs (blue triangles), and the DONET observatories (orange triangles) are shown. (**a**) The DAS experimental site is located off Shikoku Island, Japan, where the DONET observatories are deployed. (**b**) Detailed map of the Muroto cable and the surrounding area. The Muroto cable is 128 km long of which the cable section up to 50 km is used for the DAS measurement. Cable length is indicated by a blue cross at every 10 km. Two OBSs, i.e. OBS51 and OBS52, had been deployed near the Muroto cable temporally during the air-gun shots. The seismic survey lines with the air-gun shots were located over the Muroto cable and one DONET observatory, MRG27. (**c**) Detailed map of the air-gun shots near the Muroto cable. The numbers presented along two seismic survey lines, i.e. MR01 and MR02 are the shot identifications (IDs). The locations of the air-gun shot are represented by red circles every 10 shots for easier visibility. It is expected that the air-gun shots between #1670 and #1680 along the MR01 line were conducted over the Muroto cable and two OBSs. These maps were created with the Generic Mapping Tools (GMT 4.5.11, https://www.generic-mapping-tools.org) software^60^.

We conducted a unique experiment: DAS measurements contemporarily with a ship-based seismic survey using air-guns above a submarine fiber-optic cable. Thus, we report on this first opportunity to examine the in-situ DAS dataset consisting of signals originating from the active sources. The submarine cable (power and data transmission) used in the present DAS measurement had been deployed in 1997 as part of offshore earthquake and tsunami observatories^33^. We refer to this submarine cable off Muroto in Shikoku Island, Japan as the “Muroto cable” in this paper (Fig. 1). Six single mode optical fibers are encased in the submarine cable, which were used for data transmissions by the seismic and pressure sensors. Their operation stopped in 2019, and were replaced by a new permanent cabled seafloor observatory nearby, named the Dense Ocean-floor Network system for Earthquakes and Tsunamis (DONET)^34,35^. DONET and two co-located stand-alone seismometers (temporarily deployed near the submarine cable) were available during the experiment. They allowed us to compare the signals acquired by DAS with conventional geophysical sensors at the seafloor. DONET consists of two seismometers, a broadband seismometer and an accelerometer, three kinds of pressure sensors, an absolute pressure gauge (PG), a differential pressure gauge (DPG) and a hydrophone, while the stand-alone seismometer consists of a three-component geophone and a hydrophone.

In the present study, the DAS dataset acquired during the ship-based air-gun survey is examined and compared with the hydroacoustic observations by the in-situ pressure sensors. We present how the acquired DAS dataset is processed and calibrated so that it can be regarded as in-situ sensors for the detection of hydroacoustic signals, connecting the incident hydroacoustic signals with the responding seafloor motion. We also analyze ocean microseismic background noise and a small regional earthquake observed within the DAS measurements to prove that the present data processing is performed accordingly and consistent with the conventional sensors.

## Results

### DAS recording associated with hydroacoustics

A ship-based seismic survey was conducted in December 2019 to investigate the seismic structure off Honshu, Japan. Some of the survey lines were located near the DONET observatories as well as the Muroto cable (Fig. 1a). The Muroto cable is 128 km long in total, reaching 3640 m in depth (Fig. 1b). Submarine cables are assembled with various surrounding structures (internal and external mechanical strength members) for protecting them against external force depending on the deployment environments. The submarine cable is protected by an outer armor made of single or double layer of stranded steel wires to prevent stress damage in shallow water areas. Additionally, near landing stations, cables often suffer from damages by fishing tools, anchors, trawlers, and so on. For this reason, the Muroto cable has a buried cable section from 0.35 and 2.14 km distance, with a burial depth of 0.5 to 1.0 m deep below the seafloor. From the end of this section, the cable is laid and rests due to its own weight on the seafloor. The present DAS measurement was performed within a 50 km-long section from the landing station with 10-m resolution along the submarine cable. Thus, for majority of the length, the DAS measurement is performed with the submarine cable laid on the seafloor. So less coupling to the seafloor is expected compared to on-land observation where DAS measurement can be performed using buried cable. We keep in mind the DAS sensor’s sensitivity aspects due to such weak-coupling condition, when we discuss frequency characteristics of the DAS measurement using the submarine cable in this study.

**Figure 2:**
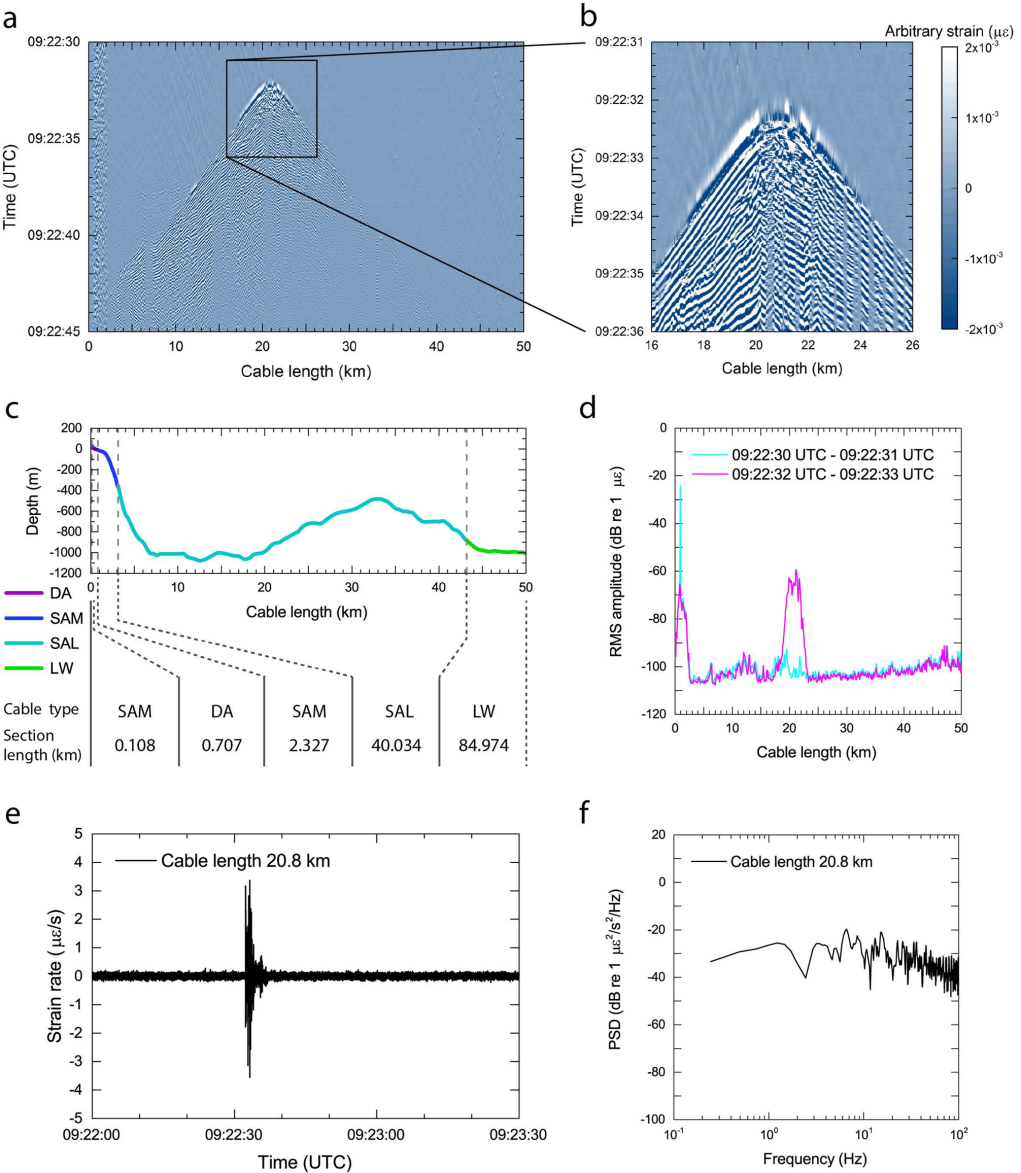
An example of the DAS recordings associated with the air-gun shot. The air-gun shot presented is number 1675 along the MR01 line indicated in Fig. 1c. (**a**) 15 s-long recording (a period for 09:22:30 UTC to 09:22:45 UTC on 03 December 2019) of strain along the Muroto cable up to 50 km. A band-pass filter between 2 and 60 Hz is applied to the strain dataset. (**b**) Enlarged figure focusing on the first arrival of the hydroacoustic signal at a cable section between 16 and 26 km. Periodic strain fluctuations are observed on both sides of the arrival. (**c**) Cross-sectional profile along the Muroto cable with information of submarine cable type. Cable types of DA, SAM, SAL, and LW mean double armored, single armored medium, single armored light, and light weight cables, respectively. (**d**) Root-mean-square (RMS) amplitudes along the Muroto cable. RMS amplitudes are calculated using 100-m bins. Two different periods between 09:22:30 UTC and 09:22:31 UTC and between 09:22:32 UTC and 09:22:33 UTC are presented. The former and the latter RMS amplitudes are associated with the ambient noise and the air-gun shot, respectively. (**e**) Time series of strain rate recording the air-gun shot at a cable length of 20.8 km. (**f**) Power spectral density (PSD) of strain rate associated with the air-gun shot.

Two seismic survey lines cross the Muroto cable at a distance of approximately 20 km, and one of the seismic survey lines (MR01) is very close to two ocean-bottom seismometers (OBS51 and OBS52) (Fig. 1c). A representative example of a DAS measurement capturing the air-gun shot signal and relevant analyses is shown in Fig. 2. This shot (Number 1675) was activated along the MR01 line at 09:22:32 UTC on 03 December 2019 near two OBSs. The horizontal offset distances between the source and OBSs are 119 m and 98 m for OBS51 and OBS52, respectively. At the same time, this shot is one of the closest sources to the Muroto cable (Fig. 1c). The raw data was converted to the strain units using the SEAFOM standard conversion formula^36,37^ and processed by applying a bandpass filter between 2 and 60 Hz to enhance the air-gun related signals.

Since the water depth beneath the ship is 852 m at the location of the shot number 1675 along the MR01 line, the first arrival of the signal, i.e. hydroacoustic signal (propagation speed 1.48 km/s) is identified 575 ms after the source initiation near the source area within the 1 km-cable section from the shot (Fig. 2a,b). The first arrival of signals near the source area is dominated by hydroacoustic signals based on its propagating speed. The Muroto cable is running almost straight in North-South direction near this source area, and therefore the arrival of hydroacoustic signals are recorded symmetrically on both sides.

The amplitude of detected signals in the landward section is larger than the seaward section for the same distance from the source location (Fig. 2a). This can be explained by a bathymetry profile along the submarine cable. The expected cross-sectional profile along the Muroto cable with cable types is provided in Fig. 2c. The Muroto cable runs across the edge of a small basin at the section between 10 km through 30 km, and then it approaches the trough axis (Fig. 1b). This bathymetry change is one possible reason why the cable section up to 30 km is relatively sensitive compared to the further seaward section. The arrival of a high-speed phase is recognized at a distance between 2 and 12 km at 09:22:37 UTC, which are refracted waves propagating through the oceanic crust. Similar phases are visible further seawards, although the amplitude is much smaller. The cable section at the location around 7 km is less sensitive for DAS measurement as indicated by the diminishing signals. This quiet characteristic was observed only during the air-gun shots, so this is attributed to the orientation of the signal source with respect to the sensor array. Another reason (in addition) could be that this cable section is located in an acoustic shadow area.

The noise level at the near-shore section up to 2 km is considerably high compared to the DAS observation on the remaining length further offshore and suggests that wind-driven waves and subsea currents continuously force oscillations of the submarine cable (Fig. 2a). Although the submarine cable is protected by double armored (DA) steel wires as well as buried near the shore line, the internal fibers were affected by the external force induced by the wind-waves. Root-mean-square (RMS) amplitudes for a period of 1 s with 100-m bins are compared in Fig. 2d; one measurement curve is taken before the air-gun shot and the other includes its signal. RMS amplitudes suggest that the noise level at the shallow water area is about 40 dB larger than the offshore area, which is as large as the air-gun shot (Fig. 2d). According to comparison of the air-gun shot recording (Fig. 2a) and the RMS amplitude (Fig. 2d), low-sensitivity sections correspond to those of the relatively low RMS amplitudes, e.g. around 7 km, 14 to 17 km, and 20 to 21 km. So DAS may contribute to assess the cable condition at the seafloor after deployment. We interpreted that the gradual increase of RMS amplitude identified further than about 30 km distance corresponds primarily to slowly increasing low-frequency wave (or subsea current) actions (Fig. 2a). The distance of 30 km is separated between the small basin slope and the offshore trough slope (Fig. 1b), so the subsea conditions may be different between these two areas.

### Data processing of DAS measurement

In principle DAS measures the phase change between an incident light pulse and the returned Rayleigh scattered light. The position of the acoustic event along the fiber-optic cable is determined by the arrival time of the returning backscatter signals. This returning backscatter phase signals are analyzed and converted to strain units representative of the axial strain in the cable induced by disturbance. Our DAS measurement system stores in real-time a continuous dataset of unwrapped phase signal over time (for each sensor channel).

Our main interest is in detecting hydroacoustic signals efficiently within a submarine cable by using the DAS technique. Thus, we examine the in-situ data of the DONET observatory during the air-gun shots. The in-situ pressure at the seafloor is proportional to the vertical acceleration of the seafloor in a frequency range where the seismic signals are prominent^38^. It is theoretically studied that the air-gun-induced pressure observed at the seafloor correlates to the particle acceleration in the shallow water where hydroacoustic waves cannot be driven^39^. The in-situ pressure observation of this study is conducted in the deep-sea and in a higher frequency range than the seismic waves, so the effect of compressible water layer arises. Hydroacoustic pressure is determined by particle velocity and acoustic impedance; this allows us to relate the in-situ pressure to the seafloor motion. Consequently, the hydroacoustic signals observed at the seafloor are proportional to particle velocity of the seafloor motion in a high frequency range where hydroacoustic signals manifested themselves (Supplementary Fig. 1). For easier comparison with conventional sensors, the dimensional unit of the DAS measurement should be converted to the particle velocity accordingly as far as hydroacoustic signals are concerned. Phase changes of the returned Rayleigh scattered light obtained by the DAS measurement are directly related to the distance changes of the fiber-optic cable in each small section. Thus, we introduce strain rate which is defined by the temporal derivative of strain.

The resulting processed data (converted to strain rate units) as induced by the air-gun shot at 09:22:32 UTC and its power spectral density (PSD) are shown in Fig. 2e,f, respectively. The waveform presents time-series data at a distance of 20.8 km where the first arrival of hydroacoustic signal was identified (Fig. 2b). A large amplitude signal train was observed at the first arrival of hydroacoustic signal, followed by a short-duration oscillation. The seismic survey line MR01 runs across the Muroto cable at a distance of 22.7 km in the sea chart (Fig. 1c), however the first arrival of the hydroacoustic signal is identified at about 1.9 km closer toward the landing station. This suggests that the actual submarine cable route and turning points near the seismic survey line MR02 has been located further south than the sea chart suggests. Although further examination may constrain the actual submarine cable route, this topic is beyond the scope of this study. The comparison with strain units of the signal of the same shot is provided in Supplementary Fig. 2. Comparing the PSD of the strain rate to the original strain, frequency contents are similar, however the amplitudes are raised-up and dropped-down in low and high frequency ranges, as an expression of the temporal derivative (Supplementary Fig. 2b). Consequently, the strain rate signal characteristics in terms of frequency contents approaches that of the hydroacoustic observations made by the standard instruments at the seafloor (Supplementary Fig. 1).

### Comparison with nearby hydrophone

**Figure 3:**
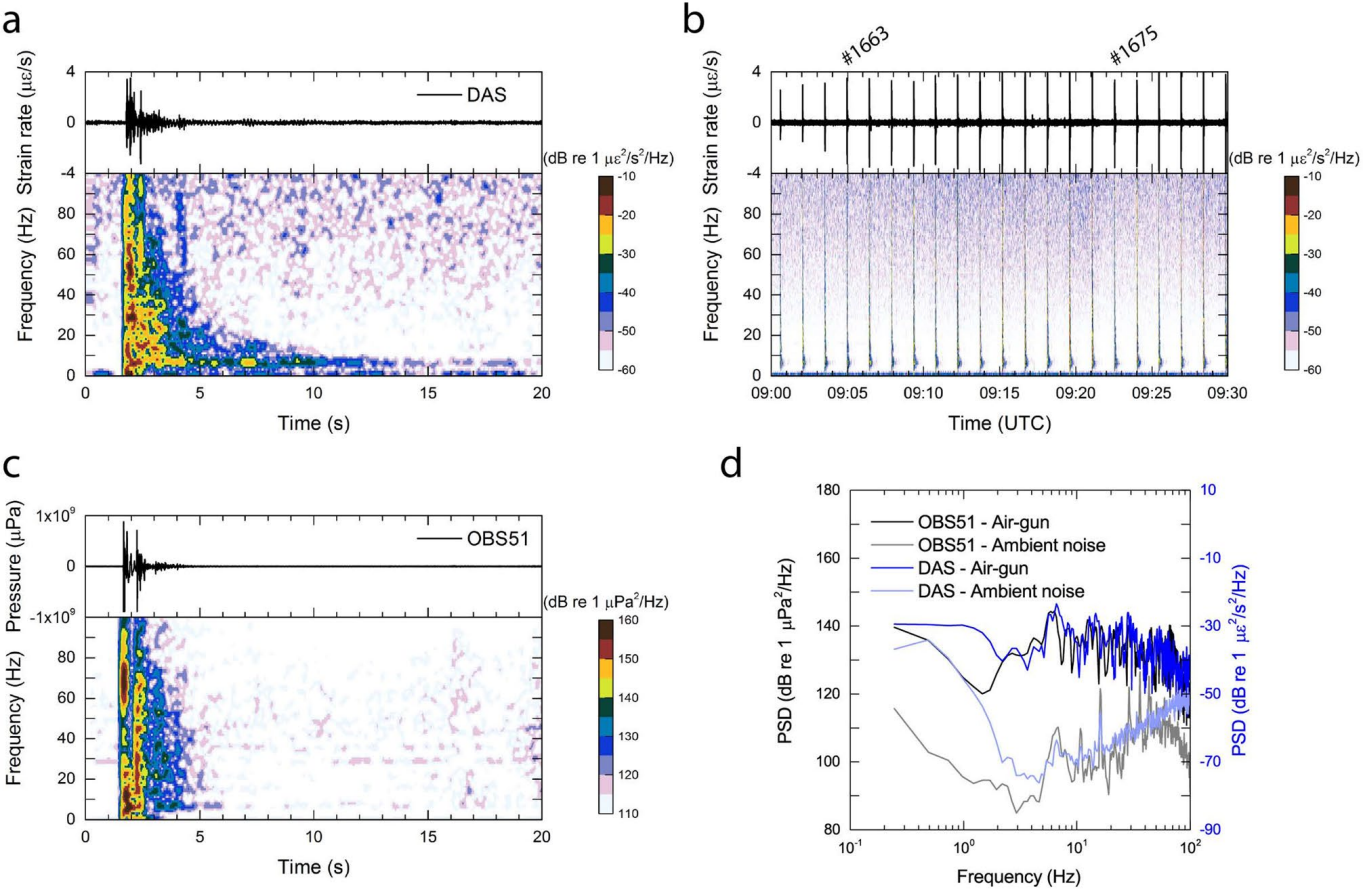
Comparison of hydroacoustic signals recorded by the DAS measurement and the co-located hydrophone. (**a**) Time series recording and its spectrogram of the DAS measurement at a cable length of 20.8 km, recording the air-gun shot number 1663 along the MR01 line. (**b**) A series of air-gun shots recorded by the DAS measurement at the same location of (**a**) at 09:00s UTC on 03 December 2019, when the ship approaching the Muroto cable and co-located two OBSs. A shot #1663 conducted at 09:04:56 UTC is magnified in (**a**). A following shot #1675 conducted at 09:22:32 UTC is also indicated. (**c**) Hydroacoustic signal originated from the same explosion of (**a**) recorded by a hydrophone of OBS51 displayed as the same form of (**a**). (**d**) Power spectral densities (PSDs) of the same signals of the DAS measurement and the hydrophone comparing with the ambient noise between the air-gun shots.

The air-gun shot (Number 1675 along the MR01 line) at 09:22:32 UTC on 03 December 2019 was the source closest to the two OBSs. Due to the uncertainty regarding the submarine cable route we assume that the location of the first arrival of the hydroacoustic signal induced by this air-gun shot is the closest point to the OBSs. Thus, we present the data at a distance of 20.8 km of the Muroto cable above (Fig. 2). Consequently we process the DAS data at that same position. Since the dynamic range of the present hydrophone (installed in the OBSs) is relatively narrow, and some of recorded signals induced by the near-field pressure wave can be clipped, we will focus on signals originated from a certain distant shot. The spectrogram of the strain rate observed at a cable sensor location of 20.8 km is shown in Fig. 3a, which is one of the successive air-gun shots blasted at 09:04:56 UTC, corresponding to the shot number 1663 along the MR01 line (Figs. 1c and  3b). The horizontal distance between the shot and the receiver is approximately 2 km. The broadband and short duration signals are identified, followed by the tail signal. Peak energy is observed in frequency range above 5 Hz.

Two OBSs, OBS51 and OBS52, were deployed within a few tens of meters (Fig. 1c); therefore there are no significant differences in recordings between them. The spectrogram of the OBS51 hydrophone recording associated with the same shot is shown in Fig. 3c. The analysis of OBS52 is provided in Supplementary Fig. 3 with the same form of Fig. 3c for further comparison. Two broadband and short-duration signals were identified, whose characteristics in the frequency-time space are similar as the DAS measurement.

PSDs associated with the same incident signal between the DAS measurement (strain rate) and the in-situ hydrophone are compared in Fig. 3d. PSDs of the ambient noise between the air-gun shots have been also calculated as a reference in order to clarify how the air-gun shot signal increases PSD in each instrument and how much is attributable to a sensor’s self-noise. Time-windows for PSDs are set to be 15 s for both the air-gun shot and the ambient noise, i.e. the first 15 s after the shot signal arrival and the last 15 s to the next shot signal arrival. A hydrophone sensor is optimized and calibrated (in contrast to DAS strain sensing technology) to observe the hydroacoustic signals in a frequency range above 1 Hz. Thus, we use it as reference for the true in-situ hydroacoustics signals. Our frequency analysis of the hydrophone suggests that the air-gun shot increases the PSD by 30 to 50 dB from the ambient noise level in a broadband frequency range. In the higher frequency range, the PSD associated with the air-gun shot signal is decreased.

The ambient noise derived from the DAS measurement does not include the signals that the hydrophone observes in a high frequency range above 5 Hz, except for a few significant, relatively low-frequency signals. The PSD of the ambient noise can be regarded as sensor self-noise because the PSD increases by a certain rate with frequency. On the other hand, DAS measures signals associated with the air-gun shots over the entire frequency range. Comparison of PSDs between the hydrophone and the DAS suggests that the DAS is capable of measuring accurately the hydroacoustic signal associated with the air-gun shots. Overall the distribution of frequency contents of the DAS is likely to follow the hydrophone at frequency range above 2 Hz. The most predominant signal of the air-gun shot is present between 5 and 10 Hz, which is demonstrated in the DAS. However, it seems that each peak of frequency contents is slightly different. The reason for this is that DAS measures the linear strain along the fiber-optic cable, whereas a hydrophone measures the hydro-pressure omni-directionally. Additionally, the DAS measured the coda waves scattered under the seafloor (Fig. 3a), while the hydrophone did not record such long-lasting waves apparently (Fig. 3c). Our analysis suggests that further investigation is needed to quantify pressure response of DAS in case of using a straightly deployed fiber-optic cable.

### Detectability of ocean microseisms

We investigate the detectability of ocean microseisms^40^ by the DAS measurement on data portions during periods of the interruption of seismic air-gun survey. Thermal effects in the fiber-optic cable interrogated by DAS cannot be neglected for such a low frequency noise analysis. There is no in-situ ground-truth data how the ambient temperature changed on the submarine cable during the present DAS measurements. It has been reported that the temperature sensors of the pressure gauges of the Muroto cable observed periodic ambient temperature changes with 0.2 °C variation in a day^41^. These temperature effects vary with fiber and cable type. One laboratory experiment suggested that the temperature coefficient impacts the fiber strain as $1\,\mu \varepsilon$ per 0.1 °C^42^, but we did not observe such large strain deviation (offset) during the data acquisition. We examined the short time duration of a few tens of seconds to an hour, and therefore we regarded that the contribution of temperature variation as very small.

**Figure 4:**
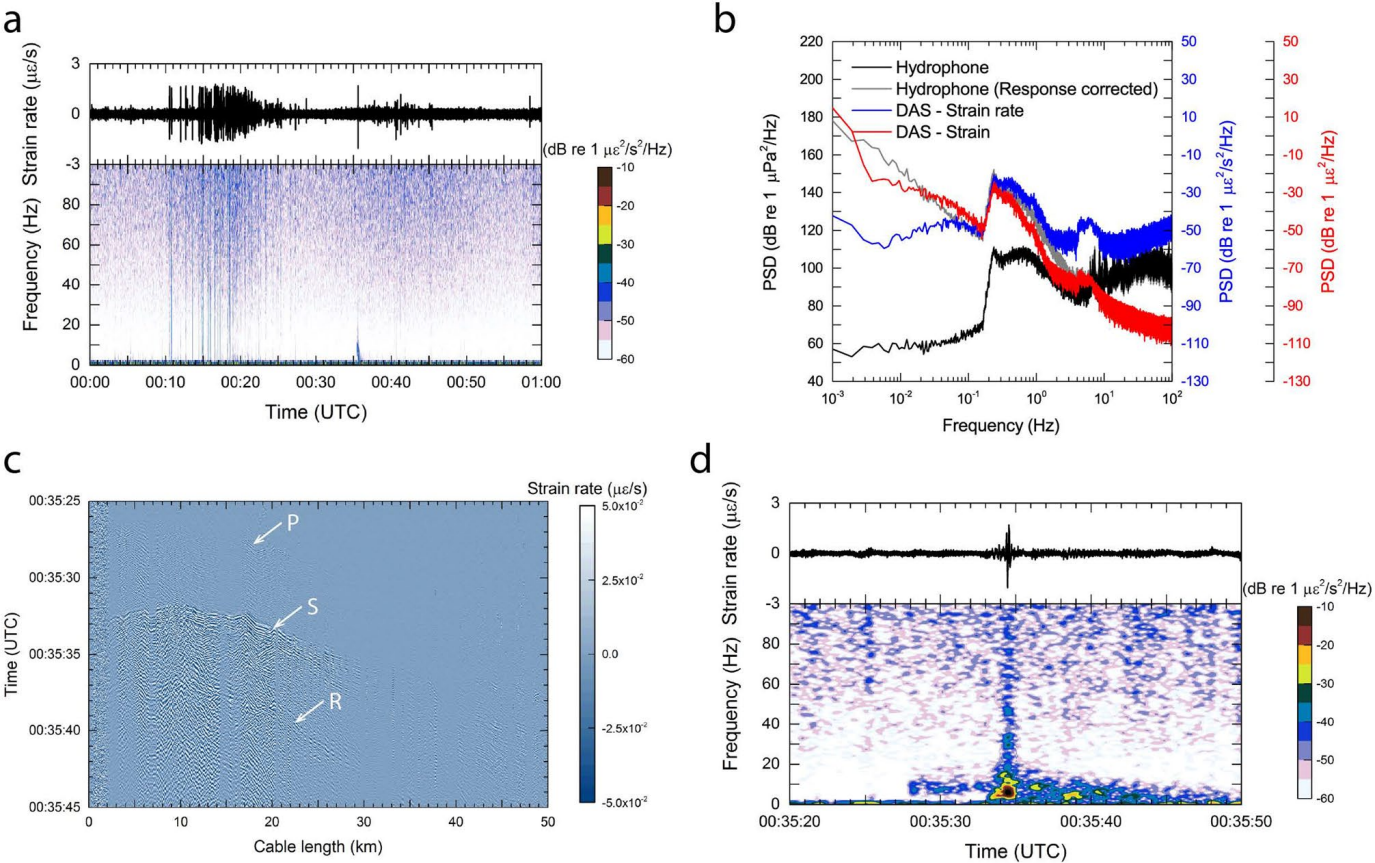
DAS recordings associated with the ambient noise and an earthquake. (**a**) Time series recording and its spectrogram of 1 h recording (a period of 00:00s UTC on 02 December 2019) at a cable length of 20.8 km. 1-s time window, 1-Hz frequency resolution and 90 % over-lap time are used. (**b**) Power spectral densities (PSDs) comparing the DAS measurement and the co-located hydrophone. Note that the PSD of the hydrophone corrected with the sensor’s response (gray line) is synthesized by extrapolation of a low frequency range down to $10^{-3}\,\text {Hz}$ using the same modeling procedure^43^. (**c**) DAS recording of the entire Muroto cable during a regional earthquake with magnitude of 1.7. Band-pass filter between 2 and 20 Hz is applied. Arrows denoted by P, S, and R represent the arrivals of seismic P-, S-, and Rayleigh- waves identified by the authors, respectively. (**d**) Time series recording and its spectrogram at a cable length of 20.8 km during the earthquake. Note that the 1-s time window is the same to (**a**), but frequency resolution and over-lap time are 0.1 Hz and 96 %, respectively.

As an example of DAS measurement of ocean microseisms, Fig. 4a shows a strain rate time series at a cable distance of 20.8 km and its spectrogram for 1-h period, i.e. 00:00s UTC on 02 December 2019. An impulsive signal having low frequency contents recognized in the spectrogram at 00:35 UTC was caused by a regional earthquake, while successive signal trains having high frequency contents during the entire period might be caused by the fishery activities nearby because of the correspondence to their working times. These signals were identified in the co-located hydrophone (Supplementary Fig. 4), supporting that strain rate is an appropriate unit to examine hydroacoustic signals in DAS measurements. The PSD of the DAS measurement of the entire period at 00:00s UTC is shown in Fig. 4b. We examined the data obtained by the co-located hydrophone of OBS51. The PSDs of the hydrophone are also shown in Fig 4b, in which the sensor response is corrected by extrapolation of frequency range down to $10^{-3}\,\text {Hz}$^43^. It shows that the hydrophone observes the ocean microseismic background noise (ocean microseisms) in the low frequency range below 1 Hz, as typically manifested in the deep ocean^40^. The PSD yields 150 dB (in pressure units) at the frequency of the ocean microseisms (Fig. 4b), which is comparable to other observations at the seafloor or in the water-column^40,44^. Since the peak frequency observed by the DAS measurement corresponds to the co-located hydrophone perfectly, it proves that the DAS is capable of observing ocean microseisms. As we discussed above, we interpreted that the sensor’s self-noise is predominant at a high frequency range above 5 Hz in the DAS measurement. The PSDs of every one-kilometer along the Muroto cable are depicted in Supplementary Fig. 5, in which the ocean microseismic background noise can be identified along the entire cable except for the near-shore section and some short-range sections.

In general, the observed pressure at the seafloor increases with decreasing frequency because the infra-gravity wave can drive long-period pressure waves in a low frequency range below $10^{-2}\, \text {Hz}$^40^. Long-period water-wave change can be regarded as static pressure change of the equivalent water-column^45^. In a borehole experiment, it is suggested that very low-frequency pressure oscillation (less than $10^{-3}\, \text {Hz}$) linearly responds to the fiber strain (displacement) measured by DAS^46^. The previous studies suggest that the DAS measurement should be processed into strain (or displacement) units, to highlight the strain induced by pressure change in the low frequency range. So we calculate PSD in strain units and plot it in Fig. 4b, in which the peak of ocean microseismic background noise is aligned between three different units. Strain units at frequency of $10^{-3}\,\text {Hz}$ increase the PSD by 40 dB higher than the peak of the ocean microseismic background noise, which is compatible with the pressure units of the hydrophone. The comparison of the DAS with the hydrophone suggests that the DAS strain measurement agrees well with the hydrophone pressure measurement at a frequency range below the ocean microseismic background noise, i.e. less than $10^{-1}\,\text {Hz}$. The present DAS measurement indicates enough sensitivity in the low frequency range down to $10^{-3}\,\text {Hz}$, and supports the possibility of detection of such long-period phase changes in a fiber-optic cable. It has been reported that a long-period strain oscillation induced by the earth tide can be detected by DAS, albeit being performed in a laboratory experiment^47^.

### Detectability of seismic signals

State-of-the-art interferometry makes it possible to detect an earthquake using thousands of kilometers of intercontinental submarine fiber-optic cable^48^. While this technique is useful for detecting an earthquake with a long distance fiber-optic cable, it has limitations due to the interferometric methodology used. It acts as a single sensor over the entire fiber-optic cable, whereas DAS provides many thousands of sensors, albeit over a shorter distance. Recently, a number of instances of on-land earthquake observations using the DAS technology have been found in the literatures^16,49,50^. However it has not been common to conduct investigations using a submarine cable; only a few field observations have been performed before^26–29^. So more applications regarding detection of earthquakes using a submarine cable are indispensable to prove that the DAS measurement can be achieved reliably in the deep-sea environment. The previous studies regarding DAS using a submarine cable addressed the far-field (epicentral distance is 16,300 km) or the regional on-land (epicentral distance varies from 40 to 100 km) earthquakes. In contrast, we focus on a regional submarine earthquake for which in-situ pressure sensors are available because we assess the effect of incident seismic signals on the DAS measurement. During the present DAS measurement, a small regional earthquake with a magnitude of 1.7 occurred at 00:35:18 UTC on 02 December 2019 at the location of 12 km north of one DONET observatory, MRA04 (Fig. 1b). The entire DAS recording in strain rate with a frequency band between 2 and 20 Hz is shown in Fig. 4c. A very weak phase is identified prior to the main phase in some cable sections, which is the seismic P wave of the earthquake. The main phase observed from 00:35:32 UTC is the seismic S wave, according to the association of epicentral distance with the seismic velocity. A weak contrast parallel to the S wave is recognized after 00:35:37 UTC, which is interpreted that the Rayleigh surface wave arrives because the time-lag from S wave increases with the epicentral distance. The azimuthal direction (bearing) of the Muroto cable changes significantly at some locations, e.g. at distances of about 10 km and about 18 km within the DAS measurement section. The incident seismic wave arrives like a spherical or a plane wave, while the Muroto cable is not straight, so such a bearing location is characterized by changing the arrival’s phase velocity and the sensitivity pattern. The P wave is attenuated beyond 18 km cable length, which corresponds to the location where the cable bearing changes from $148.4^\circ$ to $178.4^\circ$ (Fig. 1c), although the possibility that the P wave is relatively weak compared to the S wave cannot be excluded.

Hydroacoustic signals, the primary topic of this paper, are not recognized in the time-cable length plot due to two possible reasons. One possible reason is that the earthquake source is not far from the Muroto cable, so it is difficult to discriminate between seismic wave and hydroacoustic wave in this case; in other words, hydroacoustic signals might be masked by the seismic wave. The other possible reason is that the earthquake source is located near the end of the downslope, so the high-angle incident seismic wave to the water-column is not likely to be coupled to the hydroacoustic waves^51^.

A spectrogram of strain rate focusing on the earthquake signals is shown in Fig. 4d as the same form of Fig. 4a. Although the P wave arrival is not so clear, the S wave is obviously identified at 00:35:34 UTC in the spectrogram. As for the P wave, only weak signals with low frequency contents below 20 Hz are present, while for the S wave, strong signals with high frequency contents above 20 Hz are manifested. If a hydroacoustic wave originated from the earthquake source is observed, it should be exhibited at 00:35:40s UTC, based on the epicentral distance of 39 km and the hydroacoustic speed. Thus, hydroacoustic wave cannot be discriminated from the seismic wave in the case of this small earthquake. The pressure recording and its spectrogram by the DPG sensor at the DONET observatory, MRA04, is provided in Supplementary Fig. 6a, indicating that the seismic contribution to a pressure sensor is evident. The PSD indicates that the frequency contents associated with this earthquake is 20 dB in the maximum larger than the ambient noise level in the frequency range between 2 and 60 Hz (Supplementary Fig. 6b). The co-located hydrophone recording of OBS51 and its spectrogram is shown in Supplementary Fig. 7a.

When comparing PSDs, the frequency content associated with the earthquake is different between the DAS and the co-located hydrophone measurement (Supplementary Fig. 7b). One possible reason is that the signal-to-noise ratio (SNR) is quite different between the air-gun shots and the small earthquake: the pressure signal amplitude (or PSD) yields 140 dB for the air-gun shots (Fig. 3d and Supplementary Fig. 1c), while it is 100 to 120 dB for the earthquake even in the near-field observation (Supplementary Fig. 6b). Examining the ambient noise of the hydrophone (Fig. 3d) or the spectrogram before the earthquake (Supplementary Fig. 7a), the pressure amplitude varies 90 to 100 dB within frequency range of 2 to 40 Hz, which is comparable to the pressure amplitude induced by this small earthquake. The DAS measurement detected the M1.7 earthquake signals sufficiently with an increase in the PSD by 30 dB from ambient noise levels. Although further investigation may be needed, DAS using a submarine cable is better coupled to the seafloor than temporarily deployed (dropped) hydrophones. Thus, DAS has a better likelihood of detecting seismic signals. Overall the diminished SNR is confirmed in the hydrophone recording during the earthquake, however P- and S- waves arrivals can be identified in the frequency range lower than 20 Hz, apparent at 00:35:27 UTC, and are similarly recognized in the DAS measurement. Further discussion of sensitivity performed by the DAS measurement in terms of pressure response is needed. Nevertheless, the general features regarding seismic signals detection by DAS are the same to the previous studies presented elsewhere, proving that the present data processing is performed properly.

## Discussion

Hydroacoustic signals originating in the water-column or near the water-surface were detected for the first time on DAS measurements using the submarine fiber-optic cable while conducting a marine active source seismic survey. DAS measurements using a submarine cable have been studied by using the passive sources such as natural earthquake signals, while in this study active sources (ship-based air-guns) are used. Our examination of the unique dataset obtained by the DAS technique has provided insights into hydroacoustic monitoring in the ocean.

The raw dataset has been converted to strain rate along a submarine cable, so that hydroacoustics can be easily compared to standard sensors used in the present study. The sensitivity of the DAS measurement for the hydroacoustic signals induced by air-gun shots has been investigated, and PSDs show a similar characteristic in frequency contents to the co-located hydrophones. Since the sensor self-noise of the DAS measurement is relatively high in the frequency range above 5 Hz compared to other conventional in-situ hydroacoustic sensors, incident signals should have a good SNR to be detected. Nevertheless, it is worthwhile noting that the ocean microseismic background noise increases the PSD of the DAS measurement in an appropriate frequency range. This capability of the detection of the ocean miscroseismic background noise can be identified along the entire sections of the Muroto cable up to 50 km except for some short-distance sections where submarine cable may be de-coupled with the seafloor. It has been shown that seismic signals induced by a regional earthquake can be identified in the DAS measurement. Comparing with the DONET pressure sensors, some discrepancies in frequency contents and phases of the observations exist, although there is no doubt that the complete seismic signals themselves were captured.

In general, an explosion in the water-column accompanies a bubble pulse which is characterized by a short-duration and broadband frequency contents^52–54^. What is the most unique in the present study is the use of an active explosive source on a ship, and it has been demonstrated that the short-duration and broadband hydroacoustic signals were detected by the DAS measurement with very similar characteristics to the co-located hydrophones. This experimental evidence that DAS can detect hydroacoustic signals may contribute to remote monitor of submarine volcanoes. It has been well known that hydroacoustic observation associated with submarine volcanic eruptions show similar characteristics in a frequency space^55–57^. The most important aspect of hydroacoustic observation in the ocean is that the hydroacoustic signals can be driven to a distant place through the Sound and Fixed Ranging (SOFAR) channel axis^58,59^. In order to allow accurate estimation of direction-of-arrival (DOA) it is indispensable for remote monitoring of submarine volcanic activity that phase recordings at different submarine cable sections should be coherent within a few kilometers for an array analysis. It has been observed that hydroacoustic signals recorded along the Muroto cable are correlated and the phase patterns are contained within a few kilometers (Fig. 1b). Since the Muroto cable is deployed in a fairly straight line from the North to the South, some difficulties arise for application of an array analysis. The present study suggests that the DAS technology can be applied to remote monitoring of inaccessible submarine volcanic activity using hydroacoustic signals if a submarine cable route is selected accordingly.

## Methods

### The DAS system and the data processing

The DAS data acquisition system used in the present study is a long-range DAS Plexus system manufactured by OptaSense Ltd capable of recording with a sensors spacing of 1 m on a sensing length of 50 km. The raw data is acquired with 1-m channel pitch and and with a gauge length of 34 m, which corresponds to one of the gauge lengths selectable in the current DAS data acquisition system software. The gauge length was synthetically increased to 102 m by selection and summation of properly located sensor channels. The first 34 raw recorded channels were omitted during this process to avoid edge effects. After that, the raw 1-m pitch data was down-sampled to 10 m channel spacing. A data subset utilizing a 20 m gauge length was also acquired, for which a suitable summation of five properly selected sensor channels was applied to create output data with a 100 m gauge length. In both cases a very similar measurement quality was obtained. In any case, the output of the system consists of unwrapped optical phase difference data with the sampling interval of 500 Hz.

The strain $\varepsilon$ is related to the change in phase $d\phi$ by the wavelength $\lambda = 1550$ nm, refractive index of the fiber used *n*, the gauge length *G*, as individual gauge length contributions are suitably summed. In this case it results in either 102 m or 100 m dependent on the original gauge length. The photoelastic scaling factor for longitudinal strain in an isotropic material is $\xi = 0.78$. The ultimate strain is calculated by the following formula^36,37^.

1 $$\begin{aligned} \varepsilon = \frac{\lambda \, d \phi }{\; 4 \, \pi \, n \, G \, \xi \;} \end{aligned}$$

### The DONET seafloor observatory network

The dense ocean-floor network system for earthquakes and tsunamis (DONET) has been developed by the Japan Agency for Marine-Earth Science and Technology (JAMSTEC) and operated by the National Research Institute for Earth Science and Disaster Resilience (NIED). Two DONETs have been deployed off Kii Peninsula and an offshore area between Kii Peninsula and Shikoku Island. At present, 51 observatories in total are operational by two DONETs^34,35^. Each DONET observatory is connected with the fiber-optic submarine cables, and all dataset are sent to the NIED in real-time. Each observatory consists of various geophysical in-situ sensors: a three-component broadband seismometer, a three-component accelerometer, an absolute pressure gauge (PG), a difference pressure gauge (DPG), a hydrophone, and a thermometer. The sampling frequency of two kinds of seismic sensors, a DPG and a hydrophone is 200 Hz, while that of a PG is 10 Hz.

## Supplementary Information

Supplementary Information.

## Data Availability

The datasets acquired during on-site expeditions conducted by the JAMSTEC and are open to the public at the JAMSTEC repository. The datasets used in the present study are available from the corresponding author on reasonable request. The DONET data is available from the NIED at 10.17598/nied.0008.
