# Supplementary material for: Detection of hydroacoustic signals on a fiber-optic submarine cable
Source: Sci Rep. 2021 Feb 2;11:2797. doi: 10.1038/s41598-021-82093-8 (PMC7854736; doi:10.1038/s41598-021-82093-8)
Supplement: Supplementary file 1 — Supplementary Information. [file 41598_2021_82093_MOESM1_ESM.pdf]

Detection of hydroacoustic signals on a fiber-optic submarine cable

Hiroyuki Matsumoto[1], Eiichiro Araki[1], Toshinori Kimura[1], Gou Fujie[1], Kazuya Shiraishi[1], Takashi Tonegawa[1], Koichiro Obana[1], Ryuta Arai[1], Yuka Kaiho[1], Yasuyuki Nakamura[1], Takashi Yokobiki[1], Shuichi Kodaira[1], Narumi Takahashi[1,2], Robert Ellwood[3], Victor Yartsev[3], and Martin Karrenbach[3]

[1]Japan Agency for Marine-Earth Science and Technology (JAMSTEC)

[2]National Research Institute for Earth Science and Disaster Resilience (NIED)

[3]OptaSense, Inc.

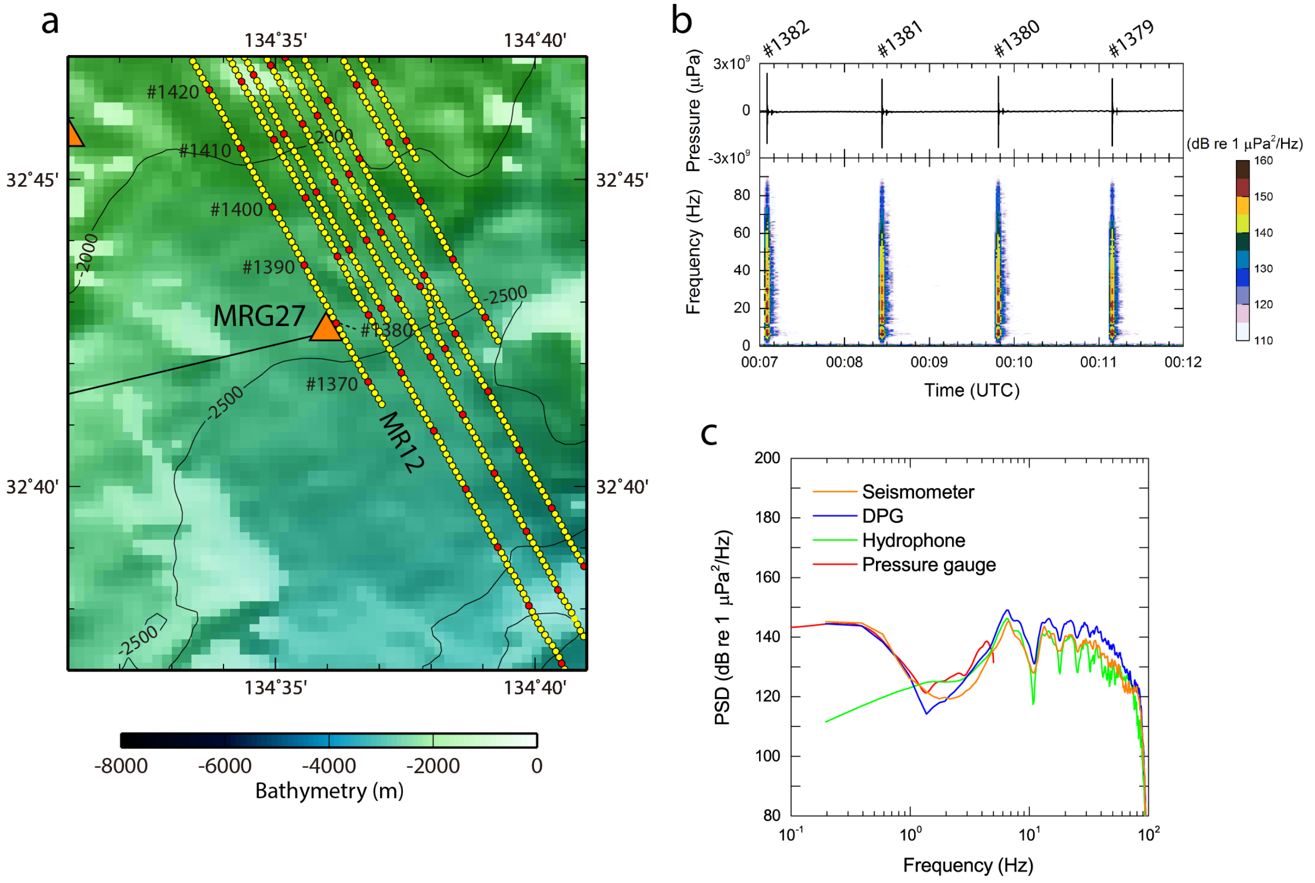

Supplementary Figure 1. (a) A map of the DONET observatory MRG27 (orange triangle) with locations of air-gun shot conducted (yellow circles). The locations at every 10 shots are represented by red circles with the shot numbers along the seismic survey line MR12. (b) Pressure waveform and its spectrogram observed by the differential pressure gauge (DPG) of MRG27. The shot numbers along the MR12 line are indicated. (c) Comparison of power spectral densities (PSDs) regarding the air-gun shot signal of a seismometer and pressure sensors (i.e. DPG, hydrophone and pressure gauge) of MRG27. This map was created with the Generic Mapping Tools (GMT 4.5.11, https://www.generic-mapping-tools.org) software[60].

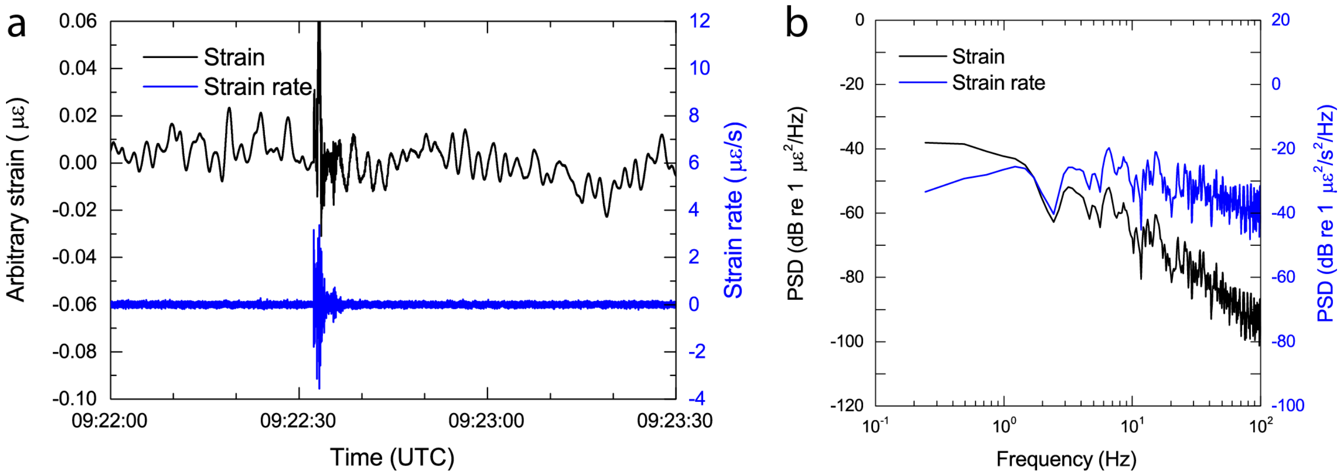

Supplementary Figure 2. (a) Waveforms and (b) power spectral densities (PSDs) of strain and converted strain rate at a cable length of 20.8 km, respectively. A de-mean value is subtracted from the raw strain data.

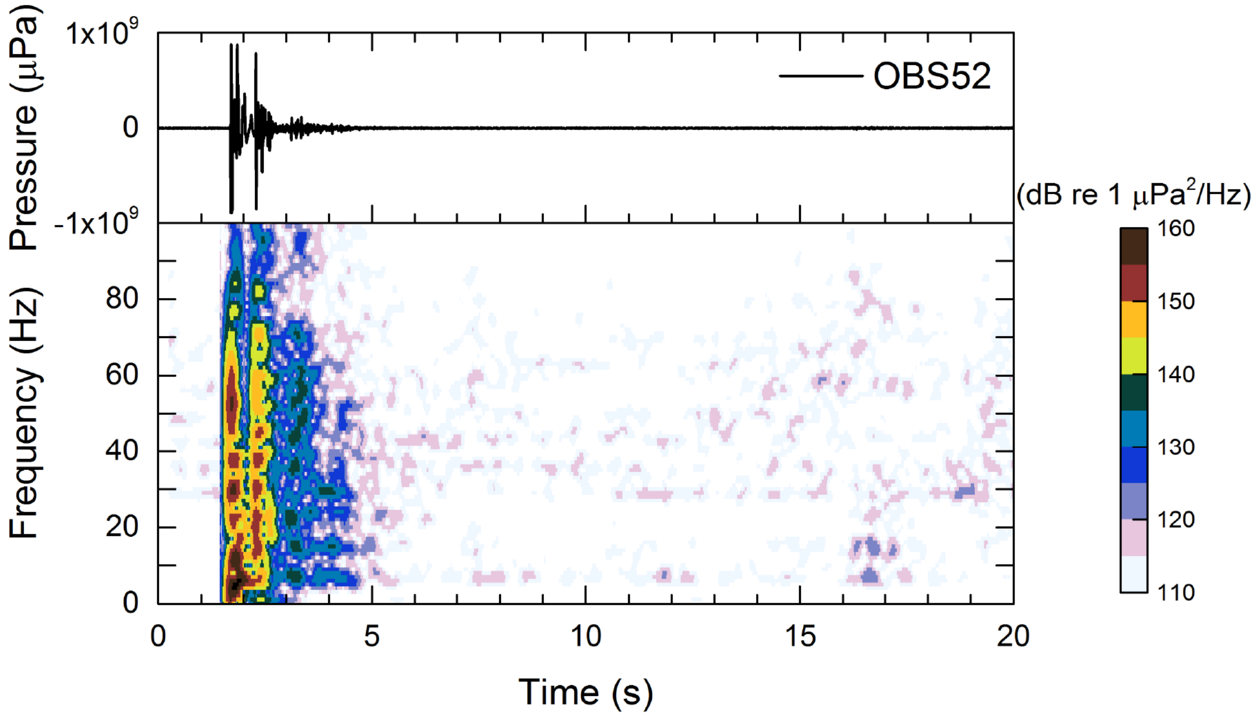

Supplementary Figure 3. Waveform and its spectrogram of a hydrophone of OBS52.

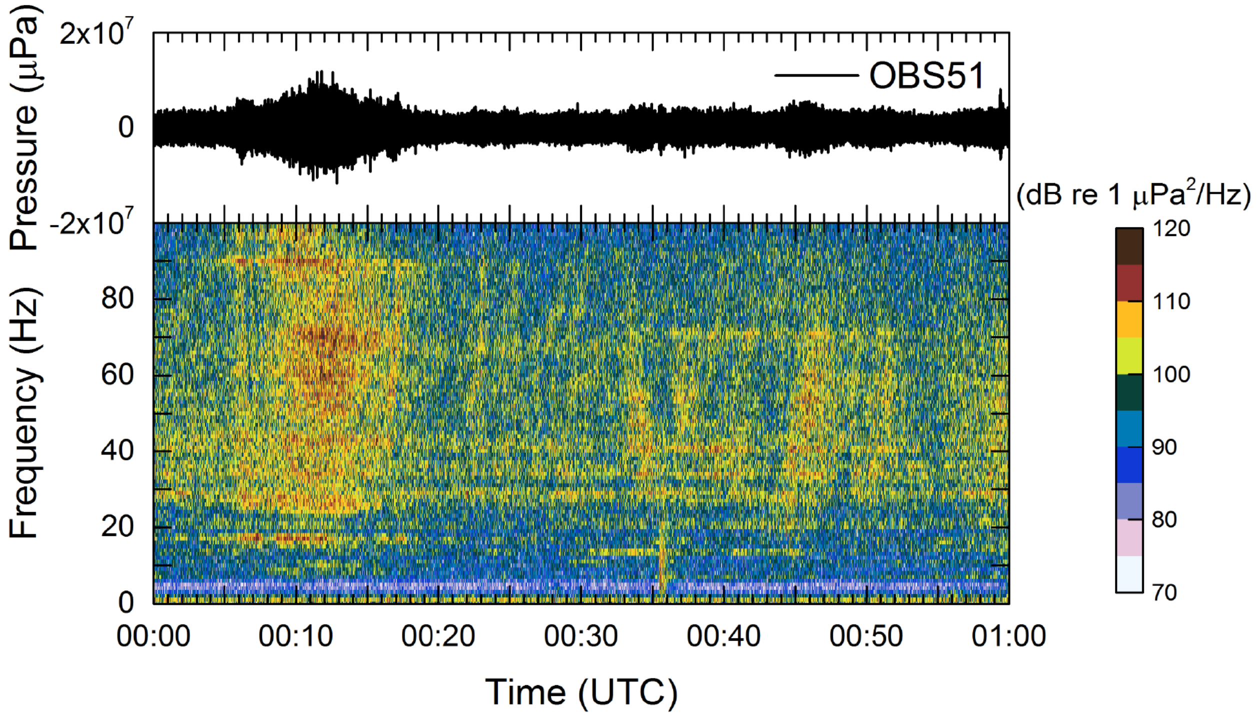

Supplementary Figure 4. Waveform and its spectrogram of a hydrophone of OBS51 for a period of 00:00s UTC on 02 December 2019.

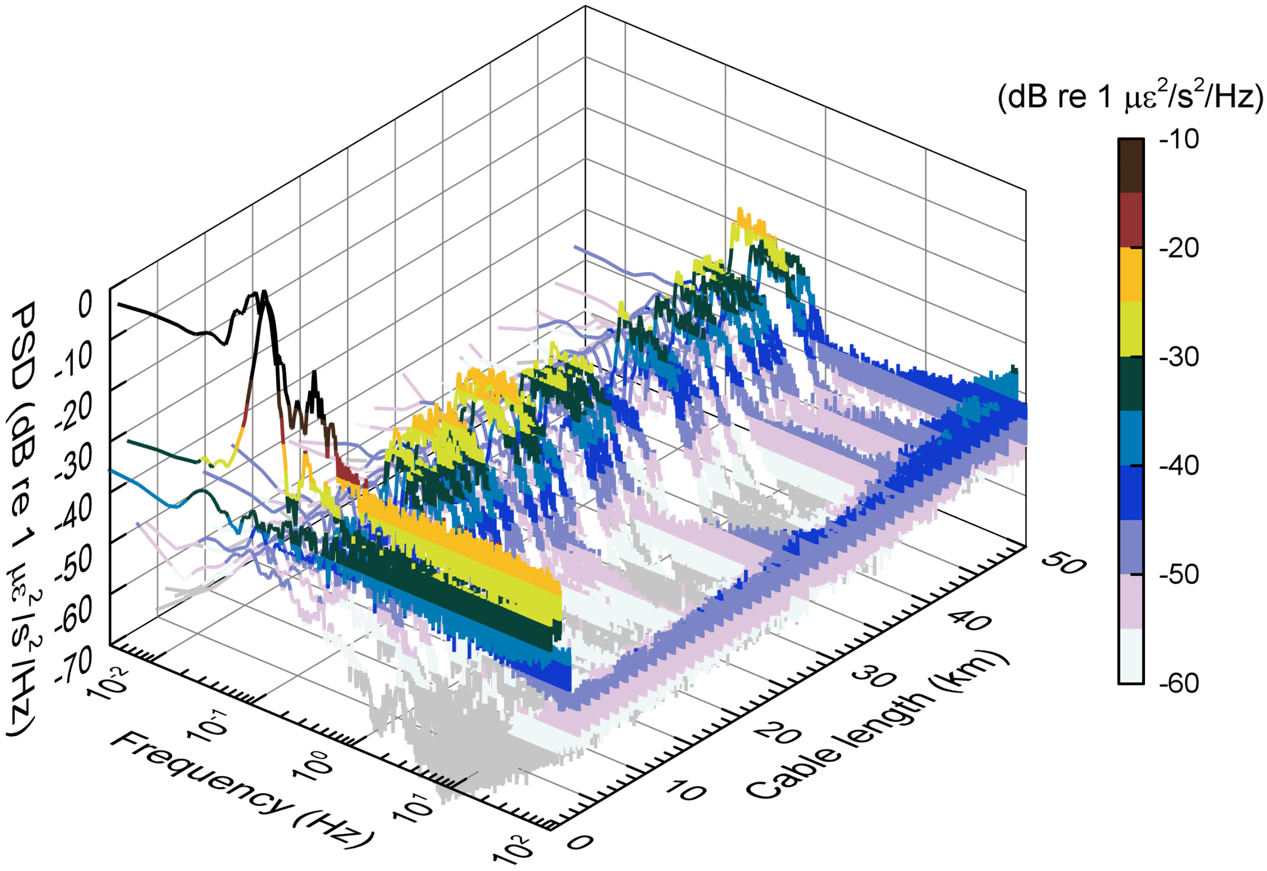

Supplementary Figure 5. Distribution of PSDs along the Muroto cable.

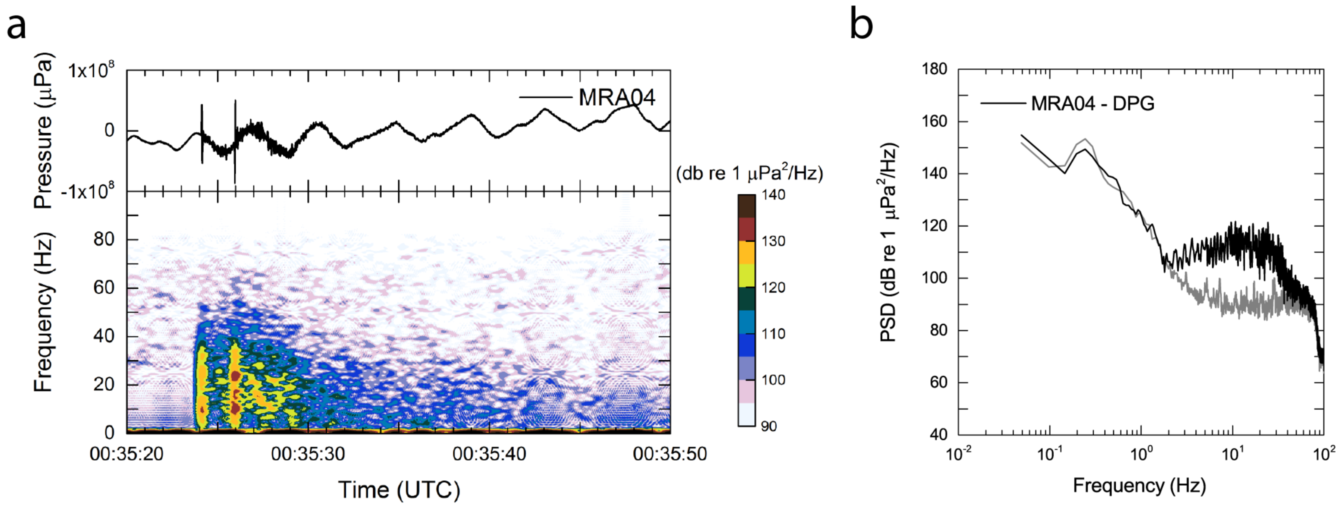

Supplementary Figure 6. (a) Pressure waveform and its spectrogram observed by DPG of MRA04 during the earthquake. (b) PSDs of DPG of MRA04 during the earthquake (black line) and the ambient noise (gray line).

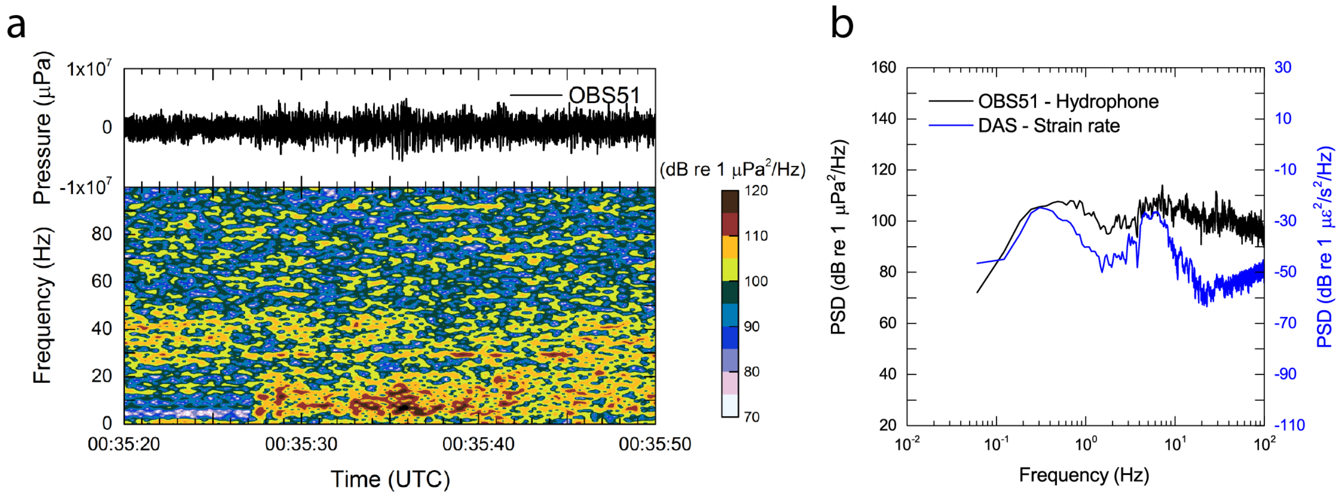

Supplementary Figure 7. (a) Pressure waveform and its spectrogram observed by the co-located hydrophone of OBS51 during the earthquake. (b) Comparison of PSDs of the DAS measurement and the co-located hydrophone..
